# Investigating the effects of exposure to extremely low frequency electromagnetic fields on job burnout syndrome and the severity of depression; the role of oxidative stress

**DOI:** 10.1002/1348-9585.12136

**Published:** 2020-07-25

**Authors:** Majid Bagheri Hosseinabadi, Narges Khanjani, Mohammad Hossein Ebrahimi, Seyed Habib Mousavi, Fereshteh Nazarkhani

**Affiliations:** ^1^ School of Public Health Shahroud University of Medical Sciences Shahroud Iran; ^2^ Environmental Health Engineering Research Center Kerman University of Medical Sciences Kerman Iran; ^3^ Environmental and Occupational Health Research Center Shahroud University of Medical Sciences Shahroud Iran; ^4^ Department of Occupational Health, Faculty of Health Mazandaran University of Medical Sciences Sari Iran

**Keywords:** catalase, electric fields, magnetic fields, malondialdehyde, maslach burnout inventory

## Abstract

**Objectives:**

This study was designed to investigate the possible effect of exposure to extremely low frequency electromagnetic fields (ELF‐EMFs) on occupational burnout syndrome and the severity of depression experienced among thermal power plant workers and the role of oxidative stress.

**Methods:**

In this cross‐sectional study, 115 power plant workers and 124 administrative personnel of a hospital were enrolled as exposed and unexposed groups, respectively, based on inclusion and exclusion criteria. Levels of oxidative stress biomarkers, including malondialdehyde (MDA), superoxide dismutase (SOD), catalase (Cat), and total antioxidant capacity were measured in serum samples. Exposure to electric and magnetic fields was measured using the IEEE Std C95.3.1 standard at each workstation. The burnout syndrome and the severity of depression were assessed using the Maslach Burnout and Beck Depression Inventory.

**Results:**

The levels of MDA and SOD were significantly lower in the exposed group than the unexposed group. The exposed group reported a higher prevalence of burnout syndrome and higher depression severity. Multiple linear regression showed that work experience, MDA level, and levels of exposure to magnetic fields are the most important predictor variables for burnout syndrome and severity of depression. In addition, a decrease in the level of Cat was significantly associated with increased burnout syndrome.

**Conclusion:**

The thermal power plant workers exposed to ELF‐EMFs are at risk of burnout syndrome and depression. These effects may be caused directly by exposure to magnetic fields or indirectly due to increased oxidative stress indices.

## 1 INTRODUCTION

The increased use of electrical and electronic devices such as home appliances and personal computers, has increased human exposure to extremely low frequency magnetic fields (ELF‐EMFs). Furthermore, some occupational groups such as power plant workers are exposed to high levels of ELF‐EMFs caused by power transmission lines compared to the general public, which is a matter of concern.^1^, ^2^ Exposure to electromagnetic fields affect brain function, hormones, and enzymatic activity, and its effect depends on the frequency and duration of exposure.^3^, ^4^ Several potential reactive mechanisms such as oscillatory resonance, and the mediating mechanism of reactive oxygen species (ROS) that induce bipolar momentum may explain the mechanism of action of these fields,^5^, ^6^, ^7^ among which, the radical pair mechanism has been more widely accepted.^8^ In this mechanism, exposure to magnetic fields can disrupt the chemical reactions associated with radical pairs by interacting with the spin of unpaired electrons of radicals, which increases the concentration of free radicals followed by inducing the potential adverse effects of exposure to very weak fields.^8^, ^9^

Increased free radicals can disrupt the balance of enzymatic and non‐enzymatic antioxidant defense systems and cause cellular damage and oxidative stress.^10^ Studies have shown that oxidative stress can lead to mitochondrial dysfunction.^11^, ^12^ Mitochondria are accountable for the maintenance of cellular homeostasis and adenosine triphosphate (ATP) production, so mitochondrial impairments cause lower ATP levels.^13^, ^14^ It has been indicated that individuals with burnout syndrome had significantly lower ATP levels.^15^ Burnout syndrome has adverse health impacts on workers’ health and well‐being and is the prime suspect of cardiovascular diseases, musculoskeletal pain, depressive symptoms, psychotropic and antidepressant treatment, job dissatisfaction and absenteeism.^16^

Exposure to magnetic fields can lead to headaches, burnout, mental distress, and exhaustion that may be due to interfering with sleep.^17^, ^18^ People suffering from burnout syndrome usually have psychosomatic, emotional, and behavioral problems such as weakness, insomnia, anxiety, depression, aggression, and irritability.^19^, ^20^, ^21^ Moreover many studies have reported the direct effect of chronic exposure to ELF‐EMFs or its indirect effects through suppressing melatonin, such as sleep disorders, anxiety, and depression; however, the results are sometimes inconsistent.^22^, ^23^ The mechanism of these effects has been postulated to be a stress response induced by exposure to magnetic fields and due to stimulation of adrenal steroid secretion.^24^ Furthermore, the overproduction of oxidative stress indices such as nitric oxide in the brain may alter the function of the hypothalamic pituitary‐adrenal axis and cause depression and anxiety.^25^, ^26^ However, the neurobehavior changes were not reported in workers inspecting transformers and distribution lines who were moderately exposed to 5.07 kV/m electric and 15.44 µT magnetic fields.^27^ This study aimed to evaluate the effect of exposure to extremely low frequency electric and magnetic fields on occupational burnout syndrome and depression severity as chronic stress‐related symptoms, and the role of oxidative stress among those who were exposed to these fields at a thermal power plant.

## 2 MATERIALS AND METHODS

The study population included all employees (n = 147) working in different parts of a thermal power plant in Semnan, Iran. The inclusion criteria were a minimum of 2 years work experience and full‐time work at the plant. The exclusion criteria were smoking, the use of antioxidant supplements such as vitamins E and C and selenium or unwillingness to participate in the study. All eligible employees were enrolled. In total, 115 workers as the exposed group and 124 hospital administrative staff were included as the unexposed group in the study. All participants in the exposed and unexposed groups were male, lived in the same geographic location at the time of the study and signed an informed consent before participating in the study. The power plant workers were mainly responsible for controlling, monitoring, inspecting, and maintaining power generating equipment. The hospital administrators were mainly in charge of organizing, budgeting, maintaining employee records and monitoring personnel performance. They had similar job tasks, and were probably exposed to similar psychosocial stressors such as interpersonal conflict and workload in their jobs. The subjects were given a demographic questionnaire prior to sampling, which included questions about age, work experience, marital status, and type of working shift.

### 2.1 Blood sampling and measurement of oxidative stress

Five milliliter of venous blood was taken from all participants. All blood samples were taken before the last working shift of the week and after at least 8‐hr fasting in both groups to assess the effects of chronic exposure to magnetic fields. After clotting, the samples were centrifuged at 5000 × g for 10 minutes to separate the serum. Serum samples were stored at −20°C until analysis. The oxidative stress biomarkersmeasured in this study included SOD, catalase (Cat) and total antioxidant capacity (TAC) as enzymes of the antioxidant system and malondialdehyde (MDA) as the most important products of lipid peroxidation. These indicators were measured using Hangzhou Eastbiopharm kits by Double Antibody Sandwich (DAS) ELISA and the mean value of three repetitions for each sample was reported. The inter‐ and intra‐assay coefficients of variation were < 12 and < 10% for all assays, respectively.

### 2.2 The measurement of electromagnetic fields

The magnetic fields were measured using Tes‐1394 (TES Electrical Electronic, Taipei, Taiwan) with a measurement range of 30‐2000 Hz and the measurement accuracy of ± 3% at 50/60 Hz, while the electric fields were measured using the HI‐3604 ELF (Holaday Industries, Eden Prairie, MN, USA) with a frequency range of 30‐2000 Hz and the dynamic range of 1 V/m to 200 kV/m in accordance with IEEE Std C95.3.1 standard and the method proposed in the studies of Cam et al.^28^, ^29^ Initially, each employee was asked to determine his working and non‐working stations (ie rest and eating) and the amount of time he spends in each station in a regular working shift. Then, measurements were made at these stations and the dose of exposure to magnetic and electric fields was calculated separately as an 8‐h time‐weighted average using the following equation:

$$\text{TWA}=\frac{\left[X_{1}\times t_{1}+\ldots +X_{n}\times t_{n}\right]}{8}$$

In this equation, “*X*” is the intensity of the magnetic flux density (μT) or the electric field strength (V/m) at the measured points and “*t*” is the amount of time that employees spent in each station. According to the standard, the device probes were placed at a height of 1 m above the ground and the measurements were made for approximately two minutes.

### 2.3 Job burnout syndrome

The Maslach Burnout Inventory‐General Survey (MBI‐GS) was used to measure occupational burnout syndrome. This questionnaire measures three aspects of occupational burnout syndrome (BS), including Emotional Exhaustion (EE), Cynicism (CY), and Professional Efficacy (PE) and contains 16 items. The participants report their own feelings about their jobs in each question using a 7‐point Likert scale from zero (never) to 6 (every day). Kalimo et al (2003), proposed an equation for calculating the final BS score which is:

BS score=0.40×EE+0.3×CY+0.3×PE

The reliability of the Persian version of MBI‐GS was reported as 0.87 in the study done by Shamloo et al^30^

### 2.4 The severity of depression

The Beck's Depression Inventory‐ll was used to assess and classify the perceived stress levels. This questionnaire is a very common tool for measuring the severity and symptoms of depression and includes 21 multiple‐choice questions. This self‐administered questionnaire is scored based on a 4‐degree Likert scale from 0 to 3.^31^ According to the study done by Ghassemzadeh et al, the Persian BDI‐II questionnaire has high internal consistency with a Cronbach's alpha of 0.87 and an acceptable test‐retest reliability (*r* = 0.74).^32^

### 2.5 Statistical analysis

The variables were summarized as frequency, percentage, mean, and standard deviation. The Kolmogorov‐Smirnov test was used to examine the normality of the data. The Chi‐square and Mann‐Whitney U tests were used to compare demographic variables, the levels of exposure to electric and magnetic fields, levels of oxidative stress, occupational burnout, and the severity of depression in the two groups. Multiple linear regression (backward) test was used to determine the predictor variables of occupational burnout and severity of depression. Demographic variables, the level of electric and magnetic field exposure and the investigated oxidative stress indices were entered into the regression model as independent variables. The non‐normal variables were normalized using the method recommended by Templetion (2011) before entering them into the model.^33^ All statistical tests were performed using SPSS v26 (IBM SPSS, IBM Corp) at the significance level of *P* < .05.

## 3 RESULTS

The demographic data of the exposed and unexposed groups are shown in Table 1. Most of the participants in the study had an age range between 30 and 40, a normal body mass index, a rotating working shift, and work experience more than 10 years. There was no significant difference between demographic characteristics of the exposed and unexposed groups.

**TABLE 1:** Demographic information of the exposed (n = 115) and unexposed (n = 124) groups

Variable	Category	Frequency (%)	exposed group (n = 115)	unexposed group (n = 124)	*P*‐value*
Age (y)	<30	51 (21.3)	17 (14.8)	34 (27.4)	0.304
	30‐40	165 (69.1)	88 (76.5)	77 (62.1)	
	>40	23 (9.6)	10 (8.7)	13 (10.5)	
BMI (kg/m^2^)**	Underweight	22 (9.2)	7 (6.1)	15 (12.1)	0.612
	Normal	165 (69.1)	83 (72.2)	82 (66.1)	
	Obese	52 (21.7)	25 (21.7)	27 (21.8)	
Work Shift	Rotating	150 (62.8)	65 (56.5)	85 (68.5)	0.228
	Fixed	89 (37.2)	50 (43.5)	39 (31.5)	
Experience (y)	>10	179 (74.9)	82 (71.3)	97 (78.2)	0.409
	≤10	60 (25.1)	33 (28.7)	27 (21.8)	
Regular exercise	Yes	88 (36.8)	47 (40.9)	41 (33.1)	0.229
	No	151 (63.2)	68 (59.1)	83 (66.9)	

* Chi‐square.

** Underweight: >18.5, normal: 18.5‐25, obese: >25

The exposure level to electrical and magnetic fields, levels of oxidative stress indices, the severity of depression, and burnout syndrome reported for the exposed and unexposed groups are presented in Table 2. Among oxidative stress indices, catalase and TAC levels, and among the occupational burnout subgroups, only the reduced professional efficiency did not show a significant difference between the two groups.

**TABLE 2:** Mean (and SD) level of exposure to electric and magnetic fields, oxidative stress indices, depression, and burnout levels in exposed and unexposed groups

Variable	Exposed group	Unexposed group	*P*‐value*
Magnetic Fields (µT)	23.8 ± 14.64	1.4 ± 0.82	<.001
Electric Fields (V/m)	24.9 ± 12.09	3.8 ± 1.75	<.001
Malondialdehyde (nmol/mL)	27.4 ± 19.79	14.9 ± 9.39	.046
Superoxide Dismutase (U/L)	33.8 ± 14.59	16.7 ± 4.22	.002
Catalase (U/mL)	182.6 ± 157.67	127.7 ± 33.64	.147
Total Antioxidant Capacity (U/mL)	2.5 ± 2.49	2.1 ± 1.09	.572
Depression severity	17.5 ± 5.99	15.1 ± 4.87	.015
Emotional Exhaustion	1.9 ± 0.88	1.2 ± 1.1	.004
Cynicism	1.9 ± 0.89	0.92 ± 0.87	<.001
Reduced Professional Efficiency	1.7 ± 1.12	1.3 ± 1.09	.062
Burnout Syndrome	1.9 ± 0.65	1.1 ± 0.75	<.001

* Mann‐Whitney U

**TABLE 3:** Results of multiple linear regression for determining the predictors of depression and burnout

Variable		β	95% Confidence Interval		*P*‐Value
Dependent	Independent		Lower	Upper	
Depression	Work Experience (>10 vs ≤10)	3.71	0.59	6.82	.021
	Malondialdehyde	0.11	0.004	0.21	.041
	Magnetic Fields	0.18	0.08	0.28	<.001
Emotional exhaustion	Work Experience (>10 vs ≤10)	0.85	0.36	1.34	.001
	Magnetic Fields	0.01	0.003	0.03	.02
Cynicism	Magnetic Fields	0.03	0.009	0.05	.007
Reduced professional efficiency	Work Experience (>10 vs ≤10)	0.71	0.18	1.24	.009
	Malondialdehyde	0.02	0.006	0.03	.007
	Catalase	−0.002	−0.004	−0.001	.033
Burnout syndrome	Work experience (>10 vs ≤10)	0.46	0.11	0.81	.01
	Malondialdehyde	0.01	0.001	0.02	.049
	Catalase	−0.002	−0.003	−0.001	.008
	Magnetic fields	0.01	0.006	0.02	.003

Exposure to magnetic fields was one of the most important predictor variables of depression severity and occupational burnout syndrome based on multiple backward linear regression. In addition, among demographic variables, increased work experience had a significant relationship with increasing severity of depression, emotional exhaustion, reduced professional efficiency, and occupational burnout syndrome.

## 4 DISCUSSION

Exposure to electromagnetic fields has increased considerably and has become inevitable due to the widespread use of electrical energy in industrial and home appliances and routinely used devices such as mobile phones. Power plant workers are one of the most important occupational groups that are significantly exposed to these fields in their work environments.

The International Agency for Cancer Research has classified these fields as possibly carcinogenic to humans. The results of this study showed a significant relation between occupational exposure to magnetic fields, occupational burnout syndrome and depression severity. Moreover it was showed that levels of some indicators of oxidative stress had increased in the exposed group which may be the possible mechanism for the effects of exposure to ELF‐EMFs on burnout and depression. We showed in previous studies that exposure to these fields disturbs the antioxidant system balance, and leads to increased levels of malondialdehyde, catalase, and superoxide dismutase, which is also associated with poor sleep quality, stress, anxiety, and depression.^18^ Thus, the present study sought to evaluate the synchronous effects of exposure to ELF‐EMFs and the elevated levels of oxidative stress biomarkers on burnout syndrome and severity of depression. Our study indicated that the severity of depression, occupational burnout (including emotional exhaustion and cynicism) and levels of lipid peroxidation and SOD were higher among employees working at the power plant which had higher exposure to magnetic fields. The third dimension of burnout, reduced professional efficiency was affected and it did decrease more in the exposed group, and it was close to significance (*P* = .062). It is likely that if the sample size was bigger, it would become significant. Based on the results of the regression model, work experience, MDA and magnetic fields were the most important predictors of depression and burnout syndrome.

Some studies have provided evidence regarding the effects of exposure to ELF‐EMFs on behavioral disorders among humans and laboratory animals. However, it is difficult to compare these studies due to differences in exposure duration and exposure intensity. Besides, most studies have been performed on laboratory animals and few studies have been done on humans. In one study, a positive association was reported between the prevalence of depression symptoms and proximity with power transmission lines among the residents and this relation was strengthened by controlling the confounding variables in the regression model.^34^ Subsequently, Wijngaarden et al (2000) found evidence with regard to the association of cumulative exposure to ELF‐EMFs and suicide due to depression among electric utility workers, especially younger workers. They attributed this issue to changes in melatonin secretion.^35^ In a recent study, a significant increase in depression and anxiety was reported among employees in the copper electrolysis unit exposed to static magnetic fields.^36^

Further studies have been conducted on behavioral disorders such as depression among laboratory animals. The exposure intensity seems to be one of the most important variables affecting the occurrence of such behaviors. Whereas no effect was seen on the brain morphology, histology, anxiety‐like, or depression‐like behaviors in chronic exposure (24 weeks) of male rats to 100 μT magnetic fields.^37^ Szemerszky et al (2010) reported that continuous exposure (24 hours a day) for 4 to 6 weeks with a relatively high intensity (0.5 mT) to magnetic fields (50 Hz) can cause depression or metabolic disturbances in adult male Sprague Dawley rats.^38^ However, when exposed to higher doses (3 mT) chronically, the incidence of anxiety‐like and depression‐like behaviors caused by increased secretion of serotonin increased, but no changes were reported in the hypothalamic‐pituitary‐adrenal axis.^24^

In addition to the direct effects of exposure to magnetic fields on increasing depression severity, studies have also reported that increased levels of malondialdehyde and cortisol have a strong relation with depression disorders.^39^, ^40^ Khajehnasiri et al (2014) showed that depression is significantly associated with decreased TAC levels and increased MDA levels. In their study, the mean severity of depression reported by male shift workers at a power plant was 11.33 (SD = 5.61), which was significantly lower than the mean severity of depression in the present study.^41^

According to the results of the present study, there was no significant difference between the level of TAC in the exposed and unexposed groups. Moreover, TAC was not a predictor of depression and burnout syndrome in the regression model. The rationale behind this may be that measurement of TAC does not provide comprehensive information about the antioxidant status, since TAC assays are not able to measure all antioxidants components and appraise the role of some significance enzymes including SOD, Cat, and glutathione peroxidase.^42^, ^43^

In our study, the power plant workers with work experience more than 10 years reported more depression, emotional exhaustion, reduced professional efficiency, and burnout syndrome compared with the workers with work experience lower than 10 years. In line with this result, some studies found that work experience is one of the most influential factors on burnout, in particular, in nurses.^44^, ^45^

In explaining the relation between exposure to magnetic fields and occupational burnout syndrome, some researchers think that exogenous stresses cause oxidative cellular stress, the formation of reactive oxygen species, reactive nitrogen species, and reactive products. These factors also cause mitochondrial metabolic dysfunction, which results in the lack of adenosine triphosphate (ATP) followed by a decrease in the cells function. This decrease in ATP levels is a detrimental factor in the occurrence of burnout syndrome. As a result, weak ambient magnetic fields (such as fields produced by transformers found in devices) and various radio frequency resonances, can increase the prevalence of burnout syndrome by increasing levels of free radicals and reactive products.^46^

Brand et al (2020) found a strong association between burnout syndrome and mitochondrial dysfunction, and showed that individuals with burnout had significantly lower ATP levels and regular physical activity had positive effects on mitochondrial activity and on symptoms of burnout and depression.^15^ In line with our study, Moragon et al reported that there was no a significant association between SOD activity and burnout or its dimensions.^47^

Since magnetic fields increase the concentration of free radicals through the RPM mechanism which is a universally accepted mechanism; consequently, burnout syndrome can also be seen among people exposed to such fields, especially power plant employees who are constantly exposed to these fields in their work environments. In addition, it has been shown that exposure to electromagnetic fields (2.45 GHz) can activate microglia.^48^ Activated microglia produce anti‐ and pro‐inflammatory cytokines and also induce superoxide production.^49^ As superoxide and nitric oxide are potent free radical precursors of toxic oxidant peroxynitrite, therefore, they act as sources of individual and combined oxidative and nitrosative damage.^50^ Maisch et al (2002) also reported improvements in the symptoms and sleep patterns of patients with chronic fatigue syndrome, with decreasing exposure to 50 Hz magnetic fields.^51^

Based on our results, we found that the power plant workers experienced high level of burnout syndrome and depression as well as increased level of some oxidative stress indices including MDA and Cat that may be due to exposure to magnetic fields. Moreover increasing work experience plays a key role in severity of burnout syndrome and depression. Although there is some evidence about the relation between exposure to magnetic fields and occupational burnout and mental health problems such as depression, but still more studies are needed in this area.

This study had some strengths and weakness. One of the strengths was that the direct and indirect effects of exposure to magnetic fields were evaluated. Furthermore, we quantified exposure to electric and magnetic fields by measuring them in different workstations during normal work shifts. However, this cross‐sectional study cannot prove causality and was conducted on only one power plant, that makes it difficult to generalize the results to all thermal power plants. We had difficulty in shift classification because workers were working in three rotating shifts (morning, evening, and night) and the unexposed group were working in two shifts (morning and evening). Thus, we could not differentiate the effect of each shift on depression and burnout syndrome.^52^ We tried to find a control group with very similar work conditions that probably had the same level of workload, similar life style and diet. But we cannot be sure that their conditions were completely similar.

## 5 CONCLUSION

Power plant workers, as one of the most important occupational groups that are widely exposed to ELF‐EMFs, and are at risk of depression and occupational burnout. These effects may be caused directly by exposure to magnetic fields or indirectly due to increased oxidative stress indices.

## DISCLOSURE

*Approval of the research protocol:* This study was performed with the approval of Shahroud University of Medical Sciences (IR.SHMU.REC.1396.64). *Informed consent:* The study objectives were explained to all participants and data was collected after obtaining written consent. *Registry and the Registration No. of the study/Trial:* N/A *Animal Studies:* N/A *Conflict of Interest:* The authors declare that they have no conflicts of interest.

## AUTHOR'S CONTRIBUTIONS

MBH and MHE contributed to the design and implementation of the research. NK and MBH performed the analysis and interpretation of the data and wrote the manuscript. SHM and FN contributed by taking samples and gathering the questionnaires. All authors read the manuscript and approved it for submission.
